# MiR-29c-3p Suppresses the Migration, Invasion and Cell Cycle in Esophageal Carcinoma via CCNA2/p53 Axis

**DOI:** 10.3389/fbioe.2020.00075

**Published:** 2020-02-20

**Authors:** Haiyong Wang, Linhai Fu, Desheng Wei, Bin Wang, Chu Zhang, Ting Zhu, Zhifeng Ma, Zhupeng Li, Yuanlin Wu, Guangmao Yu

**Affiliations:** Department of Thoracic and Cardiovascular Surgery, Shaoxing People’s Hospital (Shaoxing Hospital, Zhejiang University School of Medicine), Shaoxing, China

**Keywords:** miR-29c-3p, CCNA2, p53, esophageal carcinoma, migration, invasion, cell cycle

## Abstract

**Objective:**

In the present study, we tried to describe the role of miR-29c-3p in esophageal carcinoma (EC) and the relationship of miR-29c-3p with CCNA2 as well as cell cycle, accordingly revealing the potential molecular mechanism across cell proliferation, migration and invasion.

**Methods:**

Expression profiles of EC miRNAs and matched clinical data were accessed from TCGA database for differential and survival analyses. Bioinformatics databases were employed to predict the downstream targets of the potential miRNA, and enrichment analysis was performed on the miRNA and corresponding target gene using GSEA software. qRT-PCR was conducted to detect the expression levels of miR-29c-3p and CCNA2 mRNA in EC tissues and cells, and Western blot was performed for the examination of CCNA2, CDK1 and p53 protein levels. Subsequently, cells were harvested for MTT, Transwell as well as flow cytometry assays to examine cell viability, migration, invasion and cell cycle. Dual-luciferase reporter gene assay and RIP were carried out to further investigate and verify the targeted relationship between miR-29c-3p and CCNA2.

**Results:**

MiR-29c-3p was shown to be significantly down-regulated in EC tissues and able to predict poor prognosis. CCNA2 was found to be a downstream target of miR-29c-3p and mainly enriched in cell cycle and p53 signaling pathway, whereas miR-29c-3p was remarkably activated in cell cycle. MiR-29c-3p overexpression inhibited cell proliferation, migration and invasion, as well as arrested cells in G0/G1 phase. As suggested by dual-luciferase reporter gene assay and RIP, CCNA2 was under the regulation of miR-29c-3p, and the negative correlation between the two genes was verified. Silencing CCNA2 could suppress cell proliferation, migration and invasion, as well as activate p53 pathway, even was seen to reverse the inhibitory effect of PFTβ on p53. Besides, in the presence of low miR-29c-3p, CCNA2 was up-regulated while p53 was simultaneously inhibited, resulting in the promotion of cell migration, invasion and cell cycle arrest.

**Conclusion:**

MiR-29c-3p plays a regulatory role in EC tumorigenesis and development. MiR-29c-3p can target CCNA2 to mediate p53 signaling pathway, finally attributing to the inhibition of cell proliferation, migration and invasion, and making cells arrest in G0/G1 phase.

## Introduction

Esophageal carcinoma (EC), a common gastrointestinal neoplasm, is the fifth cause of cancer-related death in China (Wang et al., 2012). EC can be classified as esophageal squamous cell carcinoma (ESCC) and esophageal adenocarcinoma (EAC) (Zaidi and Kelly, 2017), among which ESCC is the most common histopathological type with relative high morbidity in China (Kamangar et al., 2006; Bohanes et al., 2012). At present, the main curative option of EC is surgery with adjuvant radiotherapy and chemotherapy, but the overall prognosis remains poor (Gertler et al., 2011). Therefore, studying potential molecular mechanism is of great importance for the exploration of novel therapies.

MiRNAs is a large family, and some of the miRNAs show targeted relationship with miRNAs. Genome analysis suggests that miRNA-mediated genes account for nearly 30% of the total human genome, and their expressions are firmly associated with cancers (Calin et al., 2004; Lewis et al., 2005). Studies have found that the alteration of miRNAs expression can lead to the changes of oncogenes and tumor suppressor genes, thus affecting cell proliferation, migration, invasion and apoptosis in gastrointestinal neoplasms including ESCC (Harada et al., 2016). In addition, miRNAs have been observed to be differentially expressed in ESCC as reported by multiple microarray studies. Ogawa et al. found that 22 miRNAs were up-regulated in ESCC tissues relative to that in adjacent normal tissues, whereas 4 miRNAs were down-regulated (Fu et al., 2013). Fu et al. revealed that among the 43 differentially expressed miRNAs (DEmiRNAs) found in ESCC, 27 miRNAs were decreased and the rest were increased, of which miRNA-1 was significantly reduced and attributed to the inhibition of cell proliferation, clone, migration and invasion (Yao et al., 2015). Moreover, miR-34a was found to suppress cell migration and invasion in ESCC via targeting Yin Yang-1 (Nie et al., 2015), and miR-29b was shown to function on ESCC progression through targeting MMP-2 (Qi et al., 2015).

CCNA2 (cyclin A2) is a cyclin accumulated in G1 phase and plays a regulatory role in the transitional period of G1/S and G2/M (Krasnov et al., 2017). Published literature has reported that CCNA2 functions on various cancers, like colorectal cancer (Huang et al., 2017), liver cancer (Yang et al., 2016), breast cancer (Gao et al., 2014), cervical cancer (Wu et al., 2019), and EC (Ma, 2019). In the present study, we found that miR-29c-3p was remarkably down-regulated in EC cells. Then bioinformatics methods were performed to predict the targets of miR-29c-3p, and CCNA2 was selected for further investigation, in turn evaluating the potential of miR-29c-3p/CCNA2 axis as an effective therapy for EC.

## Materials and Methods

### Bioinformatics Analysis

The miRNA and mRNA expression profiles of ESCA were downloaded from the TCGA database^1^. “edgeR” package was used to perform differential analysis, and | logFC| > 1.5 and *P-*adj < 0.01 were set as the threshold to screen out DEGs. Survival analysis was performed on DEmiRNAs to confirm the potential target miRNA. Four databases miRDB^2^, mirDIP^3^, starBase^4^, and miRTarBase^5^ were utilized to predict the targets of the miRNA, and Venn diagram was plotted to find the potential target genes. GSEA 4.0.1 software was applied to carry out enrichment analysis on the miR-29c-3p and its target gene CCNA2. According to the median expression level of CCNA2 and miR-29c-3p, EC tissue samples were divided into high (*n* = 80) and low (*n* = 80) expression groups. MSigDB^6^ was applied to access “c2.cp.kegg.v7.0.symbols.gmt” data as reference.

### Cell Culture

Human normal esophageal epithelial cell HET-1A (BNCC342346) and EC cell lines Eca-109 (BNCC337687), EC9706 (BNCC339892), KYSE150 (BNCC342590), and KYSE180 (BNCC351871) were purchased from BeNa Culture Collection (Beijing, China). All cells were grown in the Dulbecco’s Modified Eagle Medium (DMEM; Gibco, United States) supplemented with 10% fetal bovine serum (FBS; Gibco, United States), streptomycin (100 mg/mL; Gibco, United States) and penicillin (100 units/mL; Gibco, United States), and maintained in 5% CO_2_ at 37°C.

### Sample Collection

A total of 30 cases of EC tissues and matched adjacent normal tissues (2 cm in margin) were collected in the Shaoxing People’s Hospital from January 2018 to May 2019. All samples were obtained during the intraoperative period as well as firmly diagnosed by experienced pathologists, and none of the patients had received preoperative chemotherapy or radiotherapy. EC tissues separated were rapidly stored in RNA preservation solution. All procedures were performed with the approval of the Ethics Committee in the Shaoxing People’s Hospital and informed consent was obtained from all patients before this study. Patients’ clinicopathological characteristics like gender, age, histology identification results and tumor location were detailed in Table 1.

**TABLE 1:** Basic information of patients and correlation with miR-29c-3p expression.

Characteristic	Total	miR-29c-3p expression	
		Low	High
**Gender**			
Male	13	4	9
Female	17	6	11
**Age**			
<50	12	3	9
≥50	18	7	11
**Histology**			
Adenocarcinoma	5	2	3
Squamous cell carcinoma	25	8	17
**Tumor location**			
Upper esophagus	2	0	2
Middle esophagus	23	8	15
Lower esophagus	5	2	3

### Cell Transfection

For preparation, cells were grown in complete medium for at least 24 h, and washed by phosphate buffered saline (PBS; pH 7.4) before transfection. Plasmids were all purchased from GenePharma (Shanghai, China), and transiently transfected into EC cells using Lipofectamine2000 (Thermo Fisher Scientific, Inc.), consequently forming six groups of NC mimic, miR-29c-3p mimic, NC inhibitor, miR-29c-3p inhibitor, si-NC, and si-CCNA2. Transfected cells were cultured in DMEM containing 5% CO_2_ at 37°C for subsequent experiments.

### RNA Extraction and qRT-PCR

TRIzol Reagent (Invitrogen) was utilized to isolate the total RNA and Superscript II reverse transcriptase (Invitrogen) was applied for cDNA synthesis via reverse transcription using 2 μg of total samples. qRT-PCR was conducted for the detection of miR-29c-3p and CCNA2 mRNA using the Applied Biosystems 7300 Real-Time PCR System (Applied Biosystems, United States), with U6 and GAPDH as internal control. All steps were followed the manufacturer’s instructions. Primers used were designed by Sangon Biotech (Shanghai, China) as listed in Table 2. 2^–ΔΔ*Ct*^ method was used for the normalization of miR-29c-3p and CCNA2 mRNA expression levels.

**TABLE 2:** Primer sequence.

Target gene	Primer (5′–3′)
CCNA2	F: CAGAAAACCATTGGTCCCTC
	R: CACTCACTGGCTTTTCATCTTC
miR-29c-3p	F: TAGCACCATTTGAAATCGGTTA
U6	F: CTCGCTTCGGCAGCACA
	R: AACGCTTCACGAATTTGCGT
GAPDH	F: GCACCGTCAAGGCTGAGAAC
	R: TGGTGAAGACGCCAGTGGA

### Western Blot

After 48 h of transfection, cells were washed three times with cold PBS. Then proteins were extracted from cells on ice by whole cell lysate, and the concentration was assayed using BCA kit (Thermo Fisher Scientific, Waltham, MA, United States). 30 μg of the total extraction was separated through polyacrylamide gel electrophoresis (PAGE), and transferred onto the PVDF membranes (Amersham, United States) that were sequentially blocked in 5% skim milk for 1 h. Afterward, the membrane was incubated with primary rabbit polyclonal antibodies overnight at 4°C, followed by horseradish peroxidase (HRP)-labeled secondary antibody goat anti-rabbit IgG H&L (ab6721, 1:2000, Abcam, Cambridge, United Kingdom) at room temperature for 1 h. Primary antibodies contained CCNA2 (ab181591, 1:2000, Abcam, Cambridge, United Kingdom), p53 (ab32389, 1:1000, Abcam, Cambridge, United Kingdom) and GAPDH (ab9485, 1:2500, Abcam, Cambridge, United Kingdom). PBST (PBS containing 0.1% Tween-20) was utilized to wash the membranes three times following each step. Protein bands were visualized by chemiluminescence apparatus (GE, United States) and then photographed.

### MTT

Transfected cells were digested, resuspended and then plated into 96-well plates at a density of 5 × 10^3^ cells/well. At 24, 48, 72, and 96 h, 10 μL of MTT regent (5 mg/mL) was added into per well and cells were continuously cultured at 37°C for 4 h. Thereafter, the supernatant was removed and the precipitate was solubilized in 200 μL of dimethyl sulfoxide (DMSO). Neo multimode reader (Thermo Fisher Scientific) was applied to measure the absorbance at 595 nm.

### Transwell

Migration assay: Cells in logarithmic phase were placed in serum-free medium for 24 h. On the following day, cell suspension at a concentration of 2 × 10^4^ cells/mL was prepared after digestion and centrifugation. 0.2 mL of suspension was added into the Transwell inserts, and 700 μL of pre-cooled DMEM containing 10% FBS was placed out of the inserts. After 24 h of incubation in 5% CO_2_ at 37°C, unmigrated cells were wiped off with a cotton swab, while cells migrated out of the inserts were fixed by methanol for 30 min and stained in 0.1% crystal violet for 20 min. Images were captured under an inverted microscope, and five fields were randomly selected for cell count.

Invasion assay: Around 2 × 10^4^ cells were added into the upper chambers pre-coated with Matrigel matrix (Corning, NY, United States), and DMEM supplemented with 10% FBS was placed in the lower chambers. Follow-up steps were similar with migration assay as detailed above.

### Flow Cytometry

Petri dishes (6 cm) were utilized to culture transfected cells (2 × 10^5^ cells/dish) until 80% in confluence. Subsequently, cells were digested with trypsin, washed with ice-cold PBS and collected. After being fixed by 75% methanol, cells were centrifuged and suspended in RNase A (Sigma-Aldrich) followed by stained using 500 μL PI solution (Sigma-Aldrich). Flow cytometer (Beckman-Coulter) was employed to analyze the cell cycle. The percentage of cells in G0/G1, S and G2/M was calculated, respectively, and compared in each group.

### Dual-Luciferase Reporter Gene Assay

CCNA2 vectors bearing mutant and wild type 3′UTR (MUT- and WT-3′UTR) were cloned into pmiRGLO (Promega, Madison, WI, United States), forming the luciferase reporter plasmids WT-CCNA2 and MUT-CCNA2. Then the two plasmids were co-transfected with miR-29c-3p mimic or NC mimic into EC cell lines, respectively, with the Renilla luciferase expression vector pRL-TK (TaKaRa, Dalian, China) as the internal control. Cells were grown in DMEM containing 10% FBS. After 48 h, dual-luciferase detection kit (Promega, Madison, WI, United States) was utilized to examine the luciferase activity.

### RNA-Binding Protein Immunoprecipitation Assay (RIP)

Magna RIP RNA-Binding Protein Immunoprecipitation Kit (Millipore, United States) was applied in this experiment following the manufacture’s protocols. Cells were firstly lysed in RIP lysate buffer for 30 min, then the proteins obtained were incubated in RIP buffer containing magnetic beads. Ago2 antibody (ab32381, 1:50, Abcam, Cambridge, United Kingdom) was used to immune-precipitate miR-29c-3p protein complex taking normal rabbit antibody IgG (ab6712, 1:1000, Abcam, Cambridge, United Kingdom) as negative control. Consequently, protease K was utilized to purify the immunoprecipitation, and qRT-PCR was performed to detect the expression of CCNA2 mRNA.

### Statistical Analysis

SPSS 22.0 statistical software was utilized to process all data. Measurement data were expressed as mean ± standard deviation (SD). *t*-test and one-way ANOVA were employed to perform comparisons between two groups and among multiple groups, respectively. Kaplan–Meier survival analysis was conducted using the log-rank method. Pearson analysis was used to analyze the correlation between miR-29c-3p and CCNA2. Statistical significance was considered when *P* < 0.05.

## Results

### MiR-29c-3p Is Decreased in EC Tissues Accompanied by Low Survival Rate and Associated With the Increase of CCNA2

**FIGURE 1:**
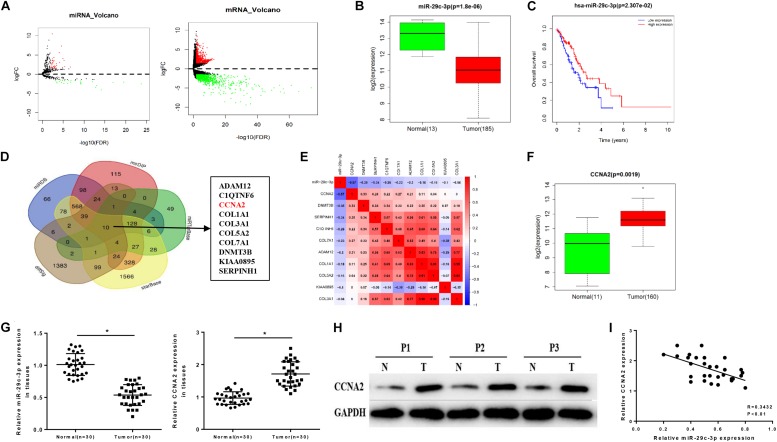
MiR-29c-3p is decreased in EC tissues accompanied by low survival rate and associated with the increase of CCNA2. TCGA database was utilized to access expression data of miRNAs and mRNAs of ESCA, and **(A)** the results of differential analysis were plotted in Volcano plots, with red representing high expression and green representing low expression. In panel **(B)**, miR-29c-3p level in EC tissues were determined as shown in a box plot. **(C)** Survival analysis of miR-29c-3p in TCGA-ESCA dataset was performed, with the red line as high expression and blue line as low expression. In panel **(D)**, Venn diagram was made to find the candidate targets of miR-29c-3p, acquiring 10 DEmRNAs. In panel **(E)**, correlation analysis was conducted between miR-29c-3p and CCNA2 (–0.57) as plotted in a heat map. In panel **(F)**, CCNA2 expression in EC cells was examined. Clinical tissue samples were used to further explore the **(G)** expression of miR-29c-3p and CCNA2 mRNA in EC tissues by qRT-PCR, **(H)** the protein level of CCNA2 (P1, P2, P3 referred to three EC samples) via Western blot and **(I)** the correlation between miR-29c-3p and CCNA2. *P < 0.05.

Differential analysis was conducted on the gene expression profiles in TCGA-ESCA dataset using “edgeR” package, acquiring 62 DEmiRNAs and 1609 DEmRNAs (Figure 1A). Among them, miR-29c-3p showed significantly low expression in EC tissues (Figure 1B). Meanwhile, survival analysis suggested that low miR-29c-3p predicted poor prognosis, showing the survival time of patients with low miR-29c-3p shorter than those with high expression (Figure 1C). In addition, miRDB, mirDIP, starBase, and miRTarBase four databases were applied to predict candidate targets of miR-29c-3p and Venn diagram was plotted to find the potential target genes. As revealed in Figure 1D, 10 DEmRNAs were obtained, among which CCNA2 presented relative high correlation with miR-29c-3p (−0.57), as well as significantly increased expression in cancer cells relative to the normal control (Figures 1E,F). GSEA suggested that miR-29c-3p was highly enriched in cell cycle, and CCNA2 was mainly activated in cell cycle and p53 signaling pathway (Supplementary Figures S1A–C).

Moreover, we detected the expression levels of miR-29c-3p and CCNA2 mRNA in clinical samples of cancer tissues by qRT-PCR, and the results showed that miR-29c-3p in EC epithelial tissues was significantly lower than that in normal tissues, while CCNA2 was highly expressed (Figure 1G). Western blot indicated that CCNA2 showed remarkably up-regulated expression in the protein level as well (Figure 1H). Besides, correlation analysis was carried out, and there was a reverse association between miR-29c-3p and CCNA2 (Figure 1I). All above results elucidated that miR-29c-3p and CCNA2 could be served as potential biomarkers for EC diagnosis.

### MiR-29c-3p Functions on the Migration, Invasion and Cell Cycle of EC Epithelial Cells

**FIGURE 2:**
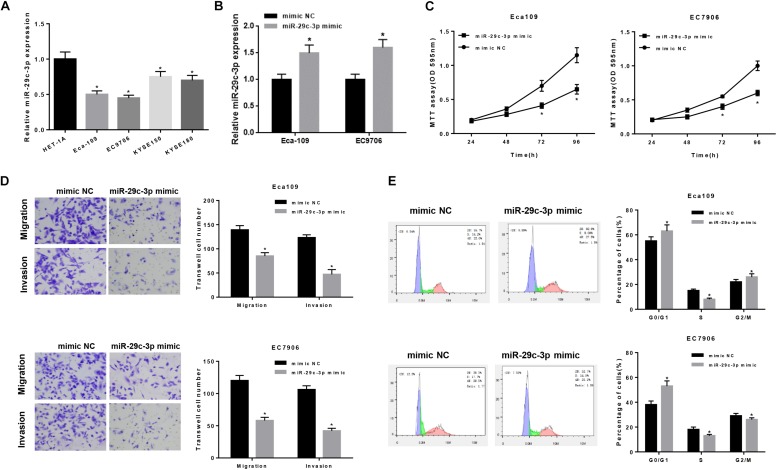
MiR-29c-3p functions on the migration, invasion and cell cycle of EC epithelial cells. qRT-PCR was performed to test **(A)** the expression of miR-29c-3p in each cell line and **(B)** that in Eca109 and EC7906 cells after transfected with miR-29c-3p mimic. To further evaluate the role of miR-29c-3p in EC cells, with the presence of miR-29c-3p mimic and normal cells as control, **(C)** cell viability was detected by MTT, **(D)** migration and invasion ability was examined by Transwell and **(E)** cell cycle was assayed by flow cytometry. *P < 0.05.

To further verify the role of miR-29c-3p in EC, qRT-PCR was firstly performed in 5 cell lines (including one normal cell line and four EC cell lines), suggesting the decreased expression of miR-29c-3p in EC cells (Figure 2A). For better observation, Eca109 and EC7906 were selected for subsequent analysis. As shown in Figure 2B, miR-29c-3p was significantly up-regulated in the two cells lines after transfection with miR-29c-3p mimic. It was also determined that cell viability was suppressed in the cells transfected with miR-29c-3p mimic as detected by MTT, indicting the weakening of cell proliferation (Figure 2C). Transwell assay revealed that EC cells had reduced migration and invasion abilities when miR-29c-3p was overexpressed (Figure 2D). Furthermore, flow cytometry showed the accumulation of miR-29c-3p mimic transfected cells in G0/G1phase (Figure 2E), indicating that miR-29c-3p could arrest cells from entering S phase, thus inhibiting cell proliferation.

### MiR-29c-3p Targets CCNA2 and Inhibits Its Expression

**FIGURE 3:**
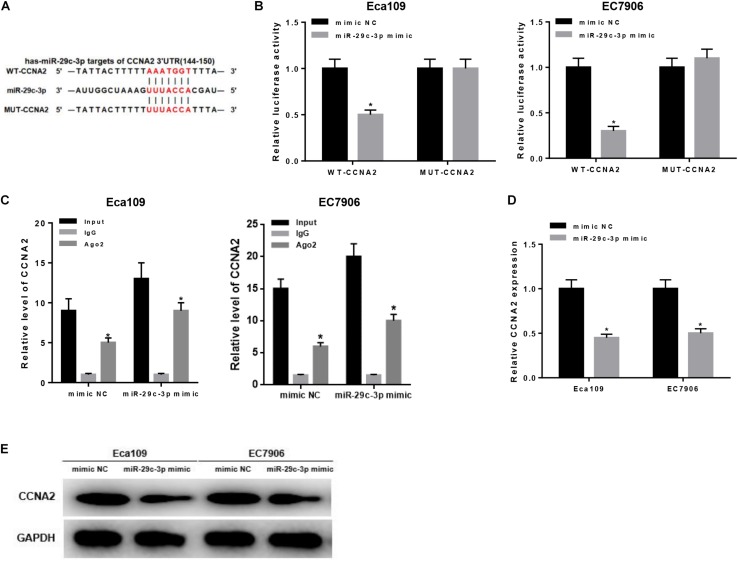
MiR-29c-3p targets CCNA2 and inhibits its expression. Targeted binding sites of miR-29c-3p and CCNA2 were predicted before as shown in panel **(A)**. To investigate their targeted relationship, **(B)** dual-luciferase assay was performed to confirm their targeted binding, and **(C)** RIP was conducted to describe the effect of miR-29c-3p on CCNA2. Moreover, **(D,E)** qRT-PCR and Western blot were carried out to determine CCNA2 expression in mRNA and protein levels in miR-29c-3p mimic transfected cells, so as to further verify such relationship. *P < 0.05.

As aforementioned, CCNA2 was a downstream target of miR-29c-3p (Figure 3A) and highly expressed in EC cells. To make a better understanding of their targeted relationship, dual-luciferase reporter gene assay was carried out via the construction of WT-CCNA2 and MUT-CCNA2. As shown in Figure 3B, luciferase activity was significantly decreased in the cells transfected with miR-29c-3p mimic and WT-CCNA2. Meanwhile, findings concluded by RIP suggested the remarkably up-regulated CCNA2 in miR-29c-3p mimic transfected cells (Figure 3C). Together, it could be elucidated that miR-29c-3p could specifically bind with CCNA2, leading to its reduced expression.

Besides, qRT-PCR and Western blot were conducted to further verify such relationship. As revealed by qRT-PCR plotted in Figure 3D, significantly reduced CCNA2 mRNA was observed when miR-29c-3p was overexpressed. Similar trend could be seen in Western blot, showing the down-regulation of CCNA2 protein level in cells transfected with miR-29c-3p mimic (Figure 3E). Collectively, there was a targeted relationship between miR-29c-3p and CCNA2.

### CCNA2 Silencing Regulates the Migration, Invasion and Cell Cycle in EC by Promoting p53 Signaling Pathway

**FIGURE 4:**
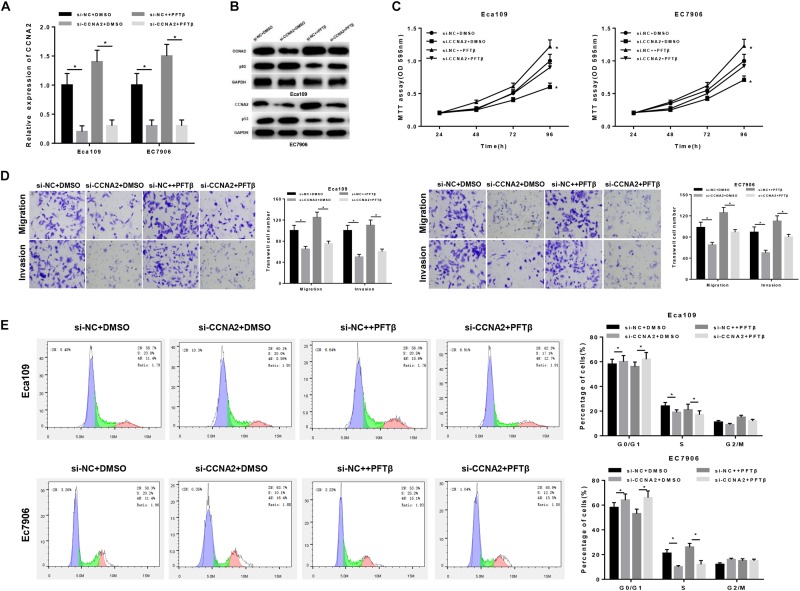
CCNA2 silencing regulates the migration, invasion and cell cycle in EC by promoting p53 signaling pathway. si-NC + DMSO, si-CCNA2 + DMSO, si-NC + PFTβ and si-CCNA2 + PFTβ were transfected into cells. qRT-PCR and Western blot were conducted to determine **(A)** the CCNA2 mRNA and **(B)** protein levels of CCNA2 as well as p53. MTT, Transwell, and flow cytometry were performed to investigate the effects of silencing CCNA2 on EC cell activities, including **(C)** cell viability, **(D)** migration and invasion, **(E)** cell cycle. *P < 0.05.

Prior studies have found that CCNA2, a type of cyclin, is able to promote cell proliferation, migration and invasion (Ma, 2019; Shekhar et al., 2019; Tu et al., 2019), as well as associated with various biological pathways like p53 signaling pathway (Zhang et al., 2018; Doan et al., 2019). In order to investigate the underlying mechanism of CCNA2 on EC cells, cells were classified into four groups: si-NC + DMSO, si-CCNA2 + DMSO, si-NC + PFTβand si-CCNA2 + PFTβ groups [PFTβ, p53 inhibitor, HY-16702, MedChemExpress, 10 μM (Da Pozzo et al., 2014)]. qRT-PCR was firstly performed to test CCNA2 level in each group, finding that CCNA2 was markedly decreased in cells transfected with si-CCNA2 + DMSO (Figure 4A). Western blot suggested that CCNA2 silencing was with a concomitant increase in p53 level. si-NC + PFTβ group presented the lowest p53 expression, while in si-CCNA2 + PFTβ group, p53 level was restored, indicating that CCNA2 silencing could abrogate the inhibitory effect of PFTβ on p53 (Figure 4B). Subsequently, transfected cells were harvested for MTT and Transwell assays, revealing that the decrease of cell viability was more prominent with the reduction of CCNA2 (Figure 4C), as well as cell migration and invasion (Figure 4D). Furthermore, flow cytometry results plotted in Figure 4E showed the accumulation of cells in G0/G1 phase with the reduction of CCNA2. Taken together, CCNA2 silencing could repress EC epithelial cell activities.

### MiR-29c-3p Mediates the Migration, Invasion and Cell Cycle in EC via CCNA2/p53 Axis

**FIGURE 5:**
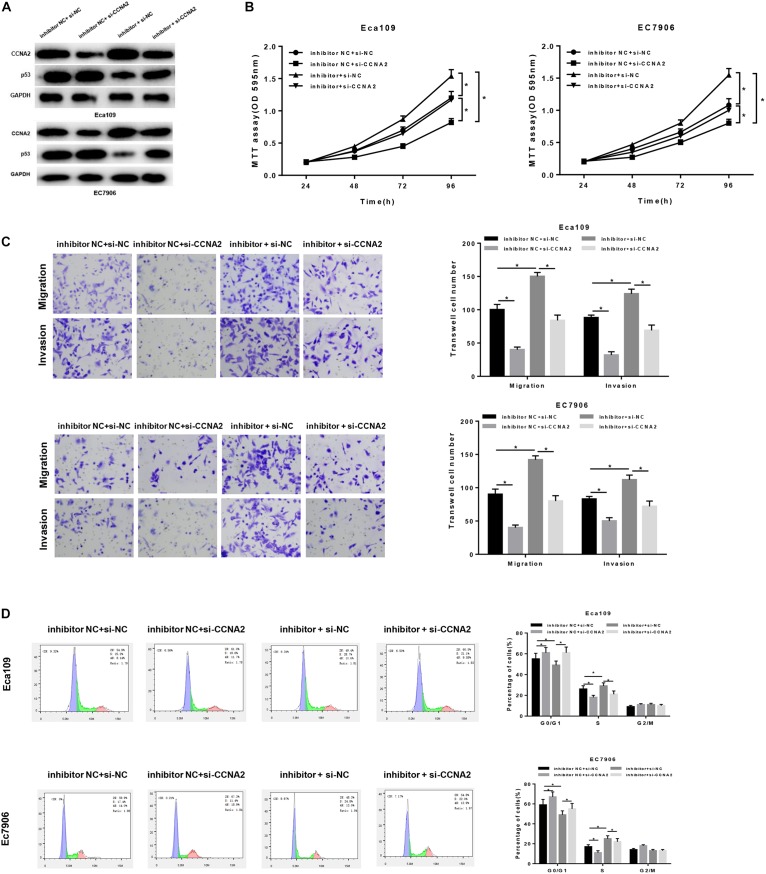
MiR-29c-3p mediates the migration, invasion and cell cycle in EC via CCNA2/p53 axis. Cells were treated with inhibitor NC + si-NC, inhibitor NC + si-CCNA2, miR-29c-3p inhibitor + si-NC and miR-29c-3p inhibitor + si-CCNA2, and then harvested for **(A)** Western blot to detect the protein levels of CCNA2 and p53. **(B)** MTT was performed to test cell viability, **(C)** Transwell was conducted to assay the ability of cell migration and invasion, and **(D)** flow cytometry was carried out to determine the effect of miR-29c-3p on cell cycle. *P < 0.05.

In order to uncover the miR-29c-3p-dependent mechanism in EC, inhibitor NC + si-NC, inhibitor NC + si-CCNA2, miR-29c-3p inhibitor + si-NC and miR-29c-3p inhibitor + si-CCNA2 were designed to transfect cells. As shown in Figure 5A, p53 expression was up-regulated when CCNA2 was silenced, whereas when miR-29c-3p was inhibited, CCNA2 was increased with a concomitant of p53 decrease. Besides, when miR-29c-3p and CCNA2 were repressed simultaneously, p53 expression was restored. Moreover, MTT assay showed that miR-29c-3p inhibitor could promote cell proliferation, while such promotive effect was inhibited when CCNA2 was silenced (Figure 5B). Similar results could be concluded as suggested by Transwell migration and invasion assays (Figure 5C). Meanwhile, with the results of flow cytometry plotted in Figure 5D, it could be indicated that the accumulation of cells in G0/G1 phase was positively associated with miR-29c-3p expression. Collectively, we speculated that miR-29c-3p targeted CCNA2 to regulate p53 signaling pathway, thereby repressing cell migration, invasion and resulting in cell cycle arrest, culminating in the inhibition of tumorigenesis.

## Discussion

Enormous evidence has revealed that miRNAs possess the ability of mediating cancer cell proliferation, migration and invasion, exerting their important regulatory roles in tumorigenesis and development (Lu et al., 2005; Deng et al., 2016). In ESCC, miR-124-3p could targeted bind with 3′UTR of BCAT1, and was found to be firmly correlated with cell proliferation and migration (Zeng et al., 2019). Renata Hezova et al. (2015) reported that miR-21, miR-29c, miR-148, and miR-203 could serve as potential diagnostic and prognostic biomarkers in EAC and ESCC. Therein, miR-29c has been reported to function on cell proliferation, migration, invasion and cell cycle, supporting its potential as a therapeutic target (Rao and Pattabiraman, 1989; Fan et al., 2014; Zhao et al., 2015; Li et al., 2018). For example, miR-29c could induce cell cycle arrest in ESCC through mediating the expression of cyclin E (Ding et al., 2011).

In the present study, we verified the low expression of miR-29c-3p in EC epithelial tissues and cells, and found that cell viability, migration and invasion were significantly decreased in cells treated with miR-29c-3p mimic. In addition, miR-29c-3p was seen to be associated with cell cycle as indicated by GSEA. Compared with the NC group, overexpression of miR-29c-3p attributed to the cell cycle arrest in G0/G1 phase, thus inhibiting the cell proliferation.

CCNA2 was found to be a direct target of miR-29c-3p, which was predicted by bioinformatics methods and verified by dual-luciferase reporter gene and RIP assays. Silencing CCNA2 could decrease the inhibitory effect of miR-29c-3p on EC epithelial cell proliferation, migration and invasion. CCNA2, a member of cyclin family, is a core regulatory factor during cell cycle progression, and participates in the regulation of S phase and mitosis. In addition, another study has showed that CCNA2 is involved in cytoskeleton dynamics behaviors and cell activities (Bendris et al., 2012), and the dysregulation of CCNA2 expression can be used as a marker of metastasis (Loukil et al., 2015). Notably, CCNA2 is commonly related to cell proliferation and highly expressed in many cancers. For instance, Cyclin A2 and Cyclin E2 can be mediated by SOSTDC1 and potentiate cell proliferation in thyroid cancer (Liang et al., 2015). FH535 can suppress cell proliferation and migration in colorectal cancer through regulating cyclin A2 and Claudin1 (Tu et al., 2019). Besides, Xu et al. (2019) found that miRNAs-mediated CCNA2 targeted p53 to inhibit cell senescence, in other words, p53/miRNAs/CCNA2 axis could be used as a novel regulator for cell senescence.

In this research, CCNA2 was found to be mainly activated in p53 signaling pathway and cell cycle detected by GSEA, and significantly up-regulated in EC tissues and cells relative to the normal controls. Silencing CCNA2 could remarkably repress cell proliferation, migration and invasion. In addition, Western blot showed that the decrease of CCNA2 attributed to the increase of p53, which played a crucial role in the regulation of cell cycle and apoptosis, as well as in the response of cell to DNA damage. When CCNA2 and p53 were simultaneously silenced, the protein level of p53 was seen to be up-regulated relative to that with p53 inhibitor alone, elucidating that silencing CCNA2 could promote p53 expression to some extent, thus activating p53 signaling pathway, consequently inhibiting cell proliferation, migration and invasion, and inducing cell arrest in G0/G1 phase.

In summary, our study confirmed that high miR-29c-3p expression can inhibit cell proliferation, migration and invasion in EC via CCNA2/p53 axis, which helps us to explore a novel approach on EC diagnosis and treatment.

## Data Availability Statement

All datasets generated for this study are included in the article/Supplementary Material.

## Ethics Statement

All procedures were performed with the approval of the Ethics Committee in the Shaoxing People’s Hospital and informed consent was obtained from all patients before this study.

## Author Contributions

HW and LF conceived and designed the study. DW, BW, CZ, and TZ performed the experiments. ZM and ZL wrote the manuscript. YW, HW, LF, and GY reviewed and edited the manuscript. All authors read and approved the manuscript.

## Conflict of Interest

The authors declare that the research was conducted in the absence of any commercial or financial relationships that could be construed as a potential conflict of interest.

## Footnotes

1 https://portal.gdc.cancer.gov/

2 http://mirdb.org/miRDB/index.html

3 http://ophid.utoronto.ca/mirDIP/index.jsp#r

4 http://starbase.sysu.edu.cn/

5 http://mirtarbase.mbc.nctu.edu.tw/php/index.php

6 http://software.broadinstitute.org/gsea/msigdb/index.jsp

## Supplementary Material

FIGURE S1 GSEA analysis of miR-29c-3p and CCNA2. **(A)** GSEA suggested that miR-29c-3p was highly activated in cell cycle. **(B,C)** GSEA revealed the correlation of high CCNA2 expression with cell cycle and p53 signaling pathway. FDR < 0.05.
