# Supplementary figures and images for: MiR-29c-3p Suppresses the Migration, Invasion and Cell Cycle in Esophageal Carcinoma via CCNA2/p53 Axis
Source: Front Bioeng Biotechnol. 2020 Feb 20;8:75. doi: 10.3389/fbioe.2020.00075 (PMC7044414; doi:10.3389/fbioe.2020.00075)

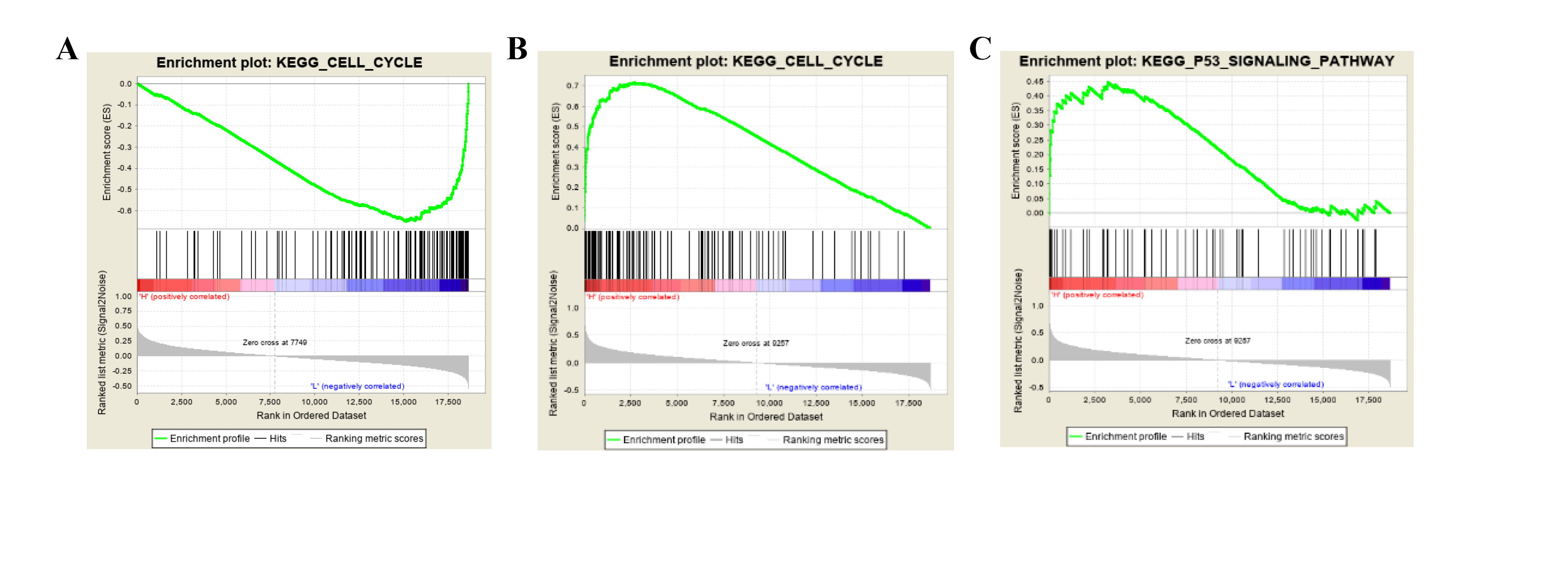

Supplement: FIGURE S1 — GSEA analysis of miR-29c-3p and CCNA2. (A) GSEA suggested that miR-29c-3p was highly activated in cell cycle. (B,C) GSEA revealed the correlation of high CCNA2 expression with cell cycle and p53 signaling pathway. FDR < 0.05. [file Image_1.tif]
